# Light‐Activated Rhenium Complexes with Dual Mode of Action against Bacteria

**DOI:** 10.1002/chem.201904689

**Published:** 2020-01-30

**Authors:** Angelo Frei, Maite Amado, Matthew A. Cooper, Mark A. T. Blaskovich

**Affiliations:** ^1^ Institute for Molecular Bioscience The University of Queensland St. Lucia Queensland 4072 Australia

**Keywords:** antibiotics, drug design, metal-based drugs, rhenium

## Abstract

New antibiotics and innovative approaches to kill drug‐resistant bacteria are urgently needed. Metal complexes offer access to alternative modes of action but have only sparingly been investigated in antibacterial drug discovery. We have developed a light‐activated rhenium complex with activity against drug‐resistant *S. aureus* and *E. coli*. The activity profile against mutant strains combined with assessments of cellular uptake and synergy suggest two distinct modes of action.

## Graphical abstract

**Shining a light on rhenium**: A light‐activated rhenium complex with activity against drug‐resistant bacteria has been developed. The activity profile against mutant strains combined with assessments of cellular uptake and synergy suggest two distinct modes of action.

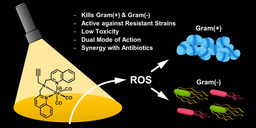

## Introduction

**Figure 1:**
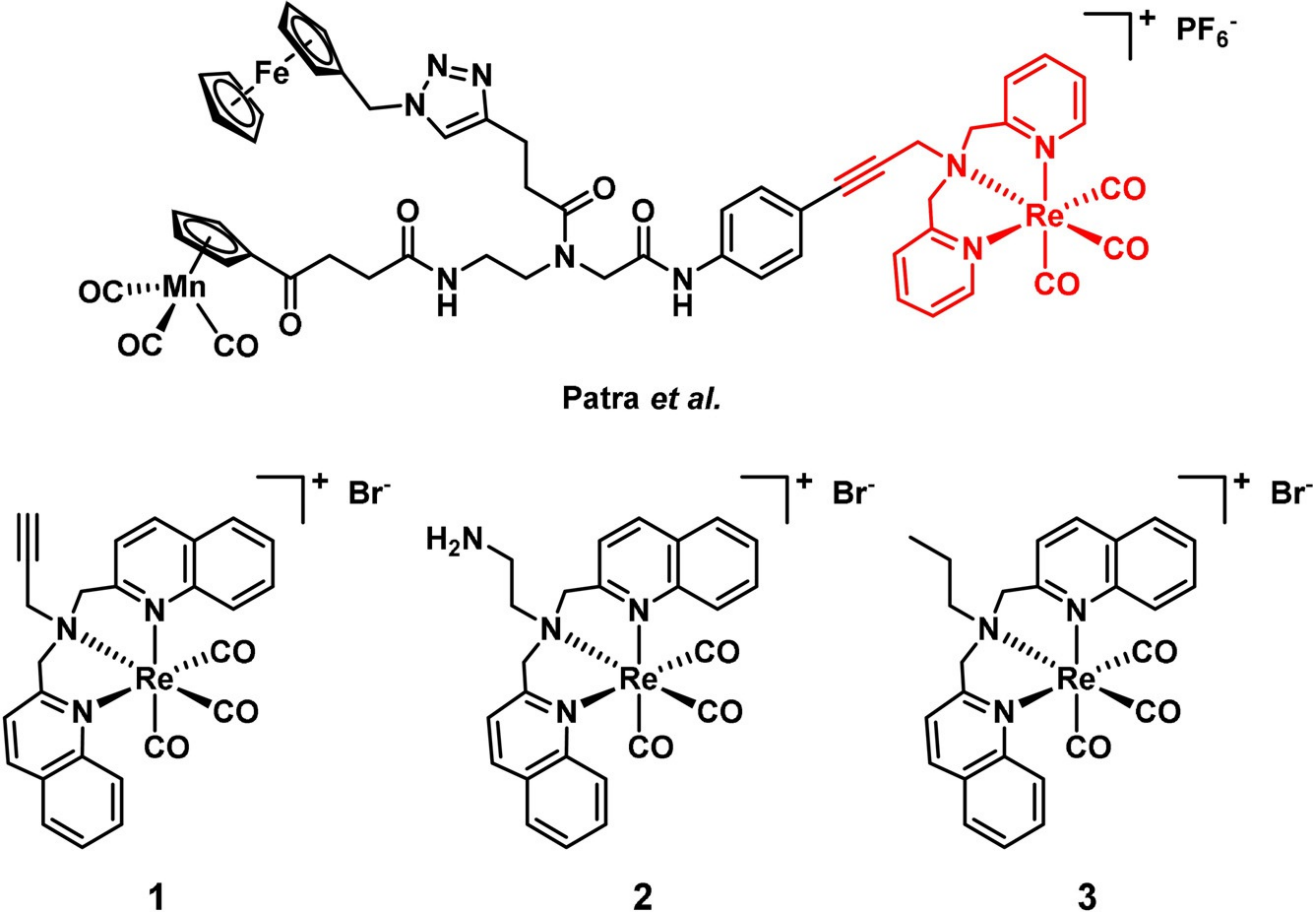
Structure of the tri‐metallic antibacterial compound reported by Patra et al.^[9]^ Structures of compounds **1**–**3** reported in this work.

The rise of widespread antimicrobial resistance has been designated by the World Health Organization (WHO) as one of the biggest threats to global health and food security.^[1]^ Most major pharmaceutical companies have shut down their antibiotic drug discovery programs, leaving academic researchers as the source of new classes of compounds, especially for the notoriously difficult to treat Gram‐negative pathogens.^[2]^ Most of these efforts focus on the investigation of purely organic compounds as antibiotics. Metal complexes provide a distinct alternative, and have proven to be promising candidates for the treatment of diseases such as malaria, Parkinson's and cancer, with several metal‐based compounds currently in clinical trials.^[3]^ However, they have only sparingly been investigated for their application against bacterial infections. In the 1950s, Dwyer and others reported on the antibacterial properties of polypyridyl metal chelates, noting that the complexes were generally more active than the metal ions.^[4]^ In the last decade, mono and oligonuclear polypyridylruthenium(II) complexes have been studied in depth by the groups of Aldrich–Wright, Keene and Collins, finding promising activity, mainly against Gram‐positive strains.^[5]^ The literature on ruthenium and other metal complexes with antibacterial activity has been summarised in recent reviews.^[6]^ In 2019 several studies on promising, highly positively charged ruthenium compounds with excellent antibacterial activity have been reported.^[7]^ Rhenium has long been overshadowed by metals such as iron and ruthenium when it comes to medicinal applications. However, there has been a continuous stream of reports on the biological applications of rhenium complexes, mainly for anticancer applications. These reports have been summarised very recently in excellent review articles by Wilson and Kühn.^[8]^ Seminal work on the antibacterial potential of rhenium compounds was reported in a series of studies led by Metzler Nolte, in which the structure–activity relationship of a tri‐metallic antimicrobial peptide and its derivatives was examined (Figure 1, top).^[9]^ The authors concluded that the [(dpa)Re(CO)_3_] moiety was crucial for the overall activity of the compound. In recent years, several other reports on rhenium complexes with some activity against Gram‐positive bacteria have been released.^[10]^

A further point of inspiration for the present work was the observation that bisquinoline rhenium tricarbonyl‐type complexes can produce reactive oxygen species (ROS), namely singlet oxygen, upon light irradiation.^[11]^ Singlet oxygen, and ROS in general, are highly reactive species that are used in photodynamic therapy (PDT) to selectively kill either cancer cells or bacteria. Given that singlet oxygen is only generated upon light irradiation, the treatment allows for spatial and temporal control of its generation, providing a targeted therapy with reduced side‐effects. Antimicrobial PDT (aPDT) has gained more attention in recent years because, unlike most antibiotics, the generated ROS do not have a specific target in the bacteria, which makes it very difficult to develop resistance against this treatment.^[12]^ Extensive studies have shown that organic cationic aPDT agents favourably target the highly negatively charged bacterial surface.^[13]^ Very recently Feng et al. reported on highly positively charged ruthenium complexes that were effective at photoinactivating MRSA while possessing low toxicity and haemolytic properties.^[7b]^

Based on the evidence that the Re(CO)_3_ core is important for non‐light‐mediated antibiotic activity, coupled with the potential for additional bactericidal activity through light‐mediated singlet oxygen generation, we sought to combine these two features. We now report the synthesis and antibacterial profiling of three rhenium bisquinoline complexes (**1**–**3**). Compounds **1**–**3** were found to possess two modes of activity against both Gram‐positive and Gram‐negative strains, including methicillin‐resistant *S. aureus* (MRSA) and colistin‐resistant *E. coli*.

## Results and Discussion

The bisquinoline scaffold was chosen as the ligand system with the goal of preparing complexes capable producing singlet oxygen upon light irradiation. The results of Patra et al. inspired us to choose an alkyne as the terminal functional group.[^9a^, ^11^] The alkyne has the added benefit of enabling further functionalisation of the system via “click”‐chemistry at a later stage. We also prepared amine (**2**) and alkyl (**3**) analogues to explore the role of the terminal functional group on the activity of the compounds. Complexes **1**–**3** were prepared by reacting ligands **L1**–**L3** (see the Supporting Information) with either Re(CO)_5_Br or [NEt_4_]_2_[ReBr_3_(CO)_3_] in MeOH under microwave irradiation for 30 min (deprotecting the amine of **2** after complexation). The compounds were purified by preparative HPLC and characterised by ^1^H and ^13^C NMR as well as by HRMS.

The antibacterial activity of the complexes was assessed by a broth microdilution minimum inhibitory concentration (MIC) assay, initially against ATCC strains of the Gram‐positive *S. aureus* and the Gram‐negative *E. coli*. To investigate the effect of light irradiation, the 96‐well plates containing bacteria and freshly added compound were irradiated with a UV lamp at 365 nm for 1 h (ca. 3 J cm^−2^) before standard overnight incubation. The UV light alone had no measurable effect on bacterial growth. In general, all three compounds showed activity with and without light against *S. aureus*, with **1** and **2** having nanomolar MIC values (Table 1 a). The MIC with light was 4‐ to 16‐fold lower than without light, indicating enhanced activity upon light irradiation for all three compounds. No activity (up to 64 μg mL^−1^) was found against *E. coli* in the absence of light. However, upon light irradiation, compound **1** gave MIC values as low as 5.8 μm (4 ug mL^−1^), with some activity also seen for compounds **2** and **3**. The control antibiotics (vancomycin for *S. aureus*, polymyxin B for *E. coli*) showed no variation in activity when exposed to light. To investigate whether **1** could also be effective against antibiotic resistant strains, we determined the MIC against both MRSA and colistin‐resistant *E. coli* (Table 1 b). Compound **1** did not show any reduction in activity against these resistant strains, suggesting that it circumvents common resistance mechanisms. To our knowledge, this makes **1** the first rhenium‐based compound that is active against both Gram‐positive and Gram‐negative pathogens, including those with inherent antibiotic resistance.

**Table 1:** MIC values of **1**–**3** against *E. coli* and *S. aureus* (a), MIC values of **1** against methicillin‐resistant *S. aureus* and collistin‐resistant *E. coli* (b), MIC values of complex **1** against various mutant strains of *E. coli* (c), and against different mutants of *P. aeruginosa* (d).

MICs against Gram(+) and Gram(−)				
*S. aureus* (ATCC25923)				
	dark		365 nm	
	[μg mL^−1^]	[μm]	[μg mL^−1^]	[μm]
Van^[a]^	1	0.7	1	0.7
1	8	11.6	0.5–1	0.72–1.45
2	32	46.2	4–8	5.8–11.6
3	2	2.9	0.25–0.5	0.36–0.72
*E. coli* (ATCC 25922)				
Pmx^[b]^	1	0.8	1	0.8
1	>64	>93.1	4–8	5.8–11.6
2	>64	>92.4	32	46.2
3	>64	>92.5	16	23.1
	000000	000000	000000	000000
MICs against resistant Gram(+) and Gram(−)				
*S. aureus* (ATCC43300; MRSA)				
Van^[a]^	2	1.4	2	1.4
1	4–8	5.8–11.6	2	2.9
*E. coli* (mcr‐1)				
Pmx^[b]^	4	3.2	4	3.2
1	>64	>93.1	8	11.6
				
MICs against mutant *E. coli* strains				
*E. coli* (MB4827; control for mutants)				
Pmx^[b]^	0.13–0.25	0.1–0.2	0.13–0.25	0.1–0.2
1	>64	>93.1	8–16	11.6–23.3
*E. coli* (MB4902; *lpxC*)				
Pmx^[b]^	0.06	0.05	0.06	0.05
1	>64	>93.1	1–4	1.5–5.8
*E. coli* (MB5747; *tolC*)				
Pmx^[b]^	0.13	0.1	0.13	0.1
1	8	11.6	2	2.9
*E. coli* (MB5746; *lpxC*, *tolC*)				
Pmx^[b]^	0.06	0.05	0.06	0.05
1	8	11.6	≤0.125	≤0.2
				
MICs against mutant *P. aeruginosa strains*				
*P. aeruginosa* (PAO1)				
Pmx^[b]^	1	0.8	1	0.8
1	>128	>186.2	64	93.1
*P. aeruginosa* (PAO397)				
Pmx^[b]^	1	0.8	1	0.8
1	128	186.2	4–8	5.8–11.6

[a] Vancomycin. [b] Polymyxin B.

Selectivity for bacterial cells over mammalian cells is critical for any potential antibiotic; hence, cytotoxicity against human cells and haemolytic properties of the rhenium complexes was assessed. Among the three complexes, **1** showed the best overall activity profile (Table S2) while showing mild toxicity against human embryonic kidney (HEK) cells with a CC_50_ of 59.9±9.2 μm, but no haemolysis up to 300 μm. In general, it is reasonable to assume that the cytotoxicity of these compounds is increased upon light irradiation. For **1**, a CC_50_ of 19.1±5.7 μm was found after irradiation with a UV lamp at 365 nm for 1 h (ca. 3 J cm^−2^). This means that even with light‐irradiation, **1** is generally more toxic against bacteria (13–26 times more effective against *S. aureus* and 1.5–3 times more effective against *E. coli*) than against human cells. Moreover, the premise of aPDT is that only areas affected by the bacterial infection are irradiated to reduce the damage to healthy cells to a minimum.

Gram‐negative bacteria are notoriously harder to kill than Gram‐positive bacteria due to the presence of an additional outer membrane, an abundance of efflux pumps, and highly selective porins that make it more difficult for compounds to reach an intracellular target.^[14]^ Indeed, compound **1** showed no activity up to 128 μg mL^−1^ against multi‐drug resistant *K. pnemoniae* either with or without light as well as only limited light activity (MIC=16–32 μg mL^−1^ and 64 μg mL^−1^) against *A. baumannii* and *P. aeruginosa* (Table 1 d and S1). We hypothesised that the lack of activity in the dark of complex **1** against the Gram‐negative strains stems from its inability to effectively accumulate inside the cells. We thus conducted MIC assays against *lpxC* and *tolC* deficient mutant *E. coli* strains, which have non‐efficient lipid A production leading to a more permeable membrane, or a deficient efflux pump, respectively.^[15]^ In general, these strains should allow for more Re‐complex to accumulate inside of the bacteria.

The *lpX* mutant resulted in a lower MIC upon light irradiation, potentially due to greater susceptibility of the mutant to the oxidative stress generated upon irradiation, but no noticeable effect in the absence of light (Table 1 c). Conversely, the *tolC* strain showed slightly improved MIC values upon irradiation but a more drastic effect without light, where the MIC decreased from >64 μg mL^−1^ (>93.1 μm) to 8 μg mL^−1^ (11.6 μm). This suggests that reduced efflux activity of the main efflux‐pump allows the compound to reach intracellular concentrations high enough for it to exhibit the same non‐light‐mediated mode of action that was previously only observed in the Gram‐positive *S. aureus*. Screening against a strain with both mutations gave a remarkably low MIC of ≤0.2 μm upon light irradiation, whereas the value remained unchanged from the *tolC* strain result in the absence of light. This further order of magnitude increase in activity with light indicates that, in this double mutant, high intracellular concentrations of **1** can be achieved and that the combination of low efflux and more permeable membrane render the strain more vulnerable to the oxidative stress generated.

**Figure 2:**
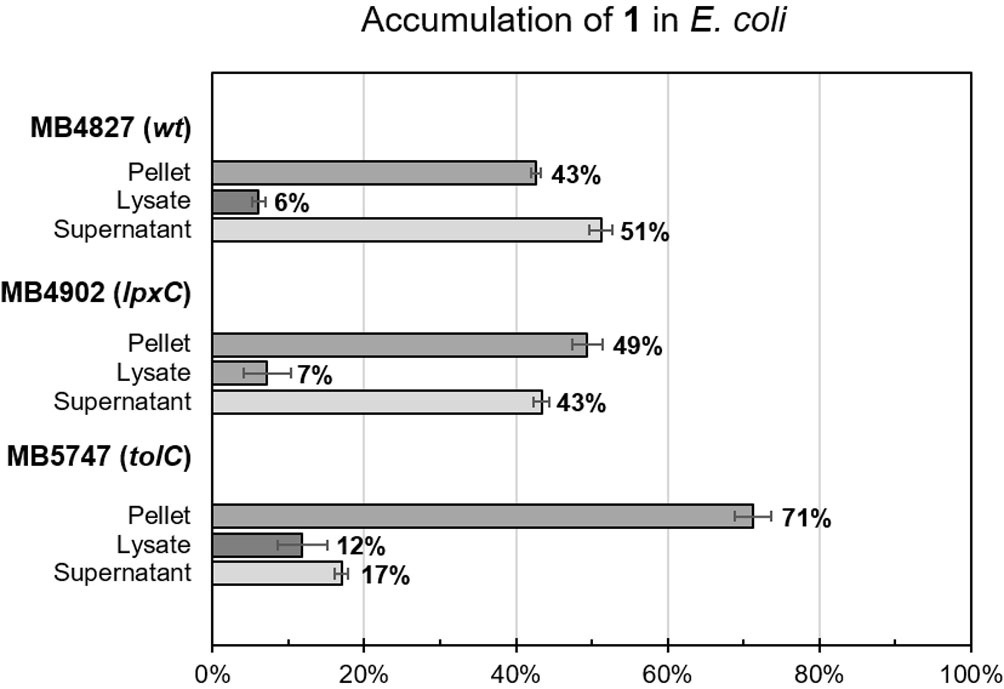
Cellular uptake and distribution of rhenium in three different *E. coli* strains after 60 min (incubated with 50 μm of **1**). Percentage of rhenium in different fractions given as percentage of total detected rhenium.

To better understand these results, we measured the cellular uptake of compound **1** to see if a correlation could be drawn between MIC and cellular accumulation. We initially attempted to adapt an assay for bacterial uptake based on fluorescence,^[16]^ but this was not successful due to overlap in the fluorescence and absorption between tryptophan and our compound. Instead, we employed inductively coupled plasma mass spectrometry (ICP‐MS) to detect the Re content. ICP‐MS is an established technique that is routinely used to determine the accumulation of metal‐based drugs into cancer cells.^[17]^ However, this technique has not yet been widely adopted for this purpose for bacteria, with one study applying an ICP‐based technique (ICP‐AES) to measure the uptake of a ruthenium complex in bacteria.^[7a]^ Another study coupled liquid chromatography and gel electrophoresis with ICP‐MS (LC‐GE‐ICP‐MS) to investigate the molecular mechanism of silver ions in bacteria.^[18]^ As with ruthenium and silver, rhenium does not occur naturally in bacteria, hence the metal can be used as a unique probe to measure the precise amount of a specific rhenium complex in a given sample. For our study, bacteria were incubated with compound **1** for either 5 or 60 min. After centrifugation, the supernatant was removed and the remaining cells (containing free intracellular or membrane/protein bound **1**) were exposed to a lysing buffer overnight. The lysate was then separated from the remaining pellet by centrifugation. After freeze‐drying and subsequent digestion in concentrated HNO_3_, the rhenium content was measured for the lysate, the remaining pellet and the initially removed supernatant. Separating the lysate and pellet allows for differentiation of complex that is “free” in the intracellular cytoplasm from complex that is bound to the membrane in some way. The cellular uptake was measured for three different bacterial strains: wild type, *lpxC*, and *tolC* mutant *E. coli*. Figure 2 shows the distribution of rhenium between the fractions in the three strains.

Overall, the apparent intracellular concentrations correlate with the measured MIC values. No significant difference in accumulation could be detected between 5 and 60 min incubation. There was a small increase in total accumulation in the *lpxC* strain compared to the wild type strain. Whereas no difference was observed in the MICs in the dark, the MIC values with light irradiation decreased from 8–16 μg mL^−1^ (control) to 1–4 μg mL^−1^ (*lpxC*). Given the natural variation of the results of MIC values and the cellular uptake data, we cannot definitively conclude that the small difference observed in the cellular accumulation between the two strains is correlated to the decrease in MIC values. A strong increase of uptake was seen in the *tolC* mutant, which mirrored the observed decrease in MIC. The percentage of rhenium found in the lysate relative to the pellet was similar for the *tolC* mutant *E. coli* and the *S. aureus* strain (Figure S1), consistent with the measured MIC values for these strains. For all the *E. coli* mutants, an increase in “free” (i.e., found in the lysate fraction) compound generally correlated with an increase in membrane‐bound complex (i.e., found in the pellet fraction). These results suggest that **1** is indeed active against *E. coli* both in the dark and with light, and further that **1** is likely a substrate for the *tolC* efflux pump and hence cannot reach concentrations high enough for its non‐light‐dependent mode of action in the wild‐type strain. This conclusion is further supported by analogous results in another Gram‐negative species, *P. aeruginosa*. In this case, **1** showed no significant activity in the dark (MIC >128 μg mL^−1^) or light irradiation (MIC=64 μg mL^−1^) in a wild‐type strain (PAO1, expressing MexAB‐OprM). Conversely, an MIC of 4–8 μg mL^−1^ was obtained with light irradiation in a multiple efflux pump deficient mutant (ΔmexAB‐oprM, ΔmexCD‐oprJ, ΔmexEF‐oprN, ΔmexJKL, ΔmexXY, ΔopmH; MIC_dark_=128 μg mL^−1^, Table 1 bottom). In this case, the uptake in the wild‐type was too low for even the light‐mediated mode of action to occur to any significant extent. In the mutant strain, a lack of efflux pump activity allowed for sufficient accumulation of **1** inside the cells for the light‐mediated activity to kill the bacteria.^[19]^

As cellular uptake seemed to be the limiting factor for the efficacy of **1**, we surmised that combining the compound with antibiotics with known effects on the integrity of bacterial membranes could potentiate its activity. To investigate this, we measured the MIC of **1** in combination with sublethal levels of the antibiotics polymyxin B, octapeptin C4, gentamicin and meropenem. Polymyxin B and octapeptin C4 both target the bacterial membrane and have been shown to increase membrane permeability, with polymyxin analogues widely used to potentiate other antibiotics.^[20]^ Gentamicin and meropenem were used as non‐membrane targeting controls, with gentamycin an aminoglycoside acting on the bacterial ribosome, and meropenem a carbapenem that inhibits bacterial cell wall synthesis.^[21]^ As seen in Table 2, **1** reaches lower (more potent) MIC values in the dark and partially with light when used in combination with the membrane targeting antibiotics, but not gentamicin and meropenem. This indicated that polymyxin and octapeptin, but not gentamicin and meropenem, increased the cellular uptake of **1**, allowing it to reach higher intracellular concentrations and hence exert both its modes of action. Interestingly, a significant increase in light‐mediated activity was observed in combination with gentamicin but not with meropenem. Possibly the oxidative stress generated by **1** synergises with the non‐lethal ribosome inhibition of gentamicin, or by the ROS effects leading to destabilisation of Fe‐S cluster and increased aminoglycoside uptake.^[22]^

**Table 2:** MIC values of **1** against *E. coli* in combination with sublethal concentrations of other antibiotics.

*E. coli* (ATCC 25922)				
	dark		365 nm	
	[μg mL^−1^]	[μm]	[μg mL^−1^]	[μm]
**1 a**	>64	>93.1	4–8	5.8–11.6
**1**+Gen^[a]^	>64	>93.1	1	1.5
**1**+Mer^[b]^	>64	>93.1	8–16	11.6–23.3
**1**+Oct^[c]^	16–32	23.3–46.5	1–2	1.5–2.9
**1**+Pmx^[d]^	16–32	23.3–46.5	4–8	5.8–11.6

[a] Gentamicin (0.25 μg mL^−1^; MIC=0.5‐2 μg mL^−1^). [b] Meropenem (0.007 μg mL^−1^; MIC=0.03–0.06 μg mL^−1^). [c] Octapeptin C4 (1 μg mL^−1^; MIC=2–4 μg mL^−1^). [d] Polymyxin B (0.03 μg mL^−1^; MIC=0.125–0.5 μg mL^−1^).

In summary, **1** is the first reported rhenium‐based compound with antibacterial activity against both Gram‐positive and Gram‐negative bacteria as well as drug‐resistant strains. We have shown that **1** has two distinct potential modes of action, one more potent activity mechanism mediated by UV‐light irradiation and a second mechanism independent of light irradiation. Although complex **1** displayed some cytotoxicity against human cells, the toxicity was 1.5–26 times higher against bacterial cells upon light irradiation. While the light‐mediated activity was demonstrated against both types of bacteria, we demonstrated that efflux pumps in the Gram‐negative strains *E*. coli and *P. aeruginosa* prevent enough accumulation of compound **1** in the cells for the light‐independent mechanism to occur. We have demonstrated that this defence mechanism can be circumvented by co‐administering **1** with sublethal concentrations of the membrane‐targeting antibiotics polymyxin B or octapeptin C4, leading to improved antibacterial activity. The dual mode of action should improve the resilience of these types of compounds against the development of resistance, and offers the prospect of an infection‐site targeted light‐based therapy. Future work will be focused on reducing the dark‐cytotoxicity of this compound class and investigating their ADMET (absorption, distribution, metabolism, and excretion) properties, which is a requirement for further development as an antibiotic. Furthermore, approaches to improve bacterial uptake of these rhenium complexes are being investigated in our lab.

## Experimental Section

**General**: All materials, unless otherwise noted, were obtained from commercial suppliers and used without further purification. [NEt_4_]_2_[ReBr_3_(CO)_3_] and Re(CO)_5_Br was kindly provided by Prof. Roger Alberto at the University of Zurich. ^1^H (600 MHz) and ^13^C (125 MHz) NMR spectra were obtained with a Bruker Avance‐600 spectrometer equipped with a TXI Cryoprobe. Chemical shifts are reported relative to the residual solvent signals in parts per million (δ). High‐resolution mass spectrometry (HRMS) was performed with a Bruker Micro TOF mass spectrometer using (+)‐ESI calibrated to NH_4_‐OAc. Analytical LC‐MS was performed with a Shimadzu LCMS‐2020 using 0.05 % formic acid in water (solvent A) and 0.05 % formic acid in acetonitrile (solvent B) as mobile phase.

LC‐MS method A: Column Zorbax Eclipse XDB‐Phenyl, 3.0×100 mm, 3.5 μm. Column temperature: 40 °C; flow rate: 1 mL min^−1^; gradient timetable: 0.00 min, 5 % B; 0.50 min, 5 % B; 3.00 min, 100 % B; 4.2 min, 100 % B; 5.00 min, 5 % B. HPLC purification was performed with a Gilson PLC 2020 system using water (solvent A) and acetonitrile (solvent B) as mobile phase.

HPLC method A: Column XTerra Prep RP18 OBD 5 μm, 19×100 mm. Flow rate: 20 mL min^−1^. Gradient timetable: 0.00 min, 10 % B; 22.00 min, 80 % B; 24.00 min, 80 % B; 25.00 min, 10 % B; 27.00 min, 10 % B. All final products were >95 % pure as determined by LC‐MS using UV at 254 nm, ELSD and APCI/ESI‐MS.

**General procedure for rhenium complexes**: The rhenium complexes **1**, **2** and **3** were prepared from either Re(CO)_5_Br or [NEt_4_]_2_[ReBr_3_(CO)_3_]. Ligand (**L1**, **L2** or **L3**, 1 equiv.) was dissolved in MeOH (3 mL) in a Biotage microwave vial (2–5 mL). The respective rhenium precursor was added and the solution was heated in a microwave to 120 °C for 30 min ([NEt_4_]_2_[ReBr_3_(CO)_3_]) or 60 min (Re(CO)_5_Br). The solvent was evaporated and the crude product was re‐dissolved in a 1:1 CH_3_CN/H_2_O mixture and purified by preparative HPLC (method A). The complexes were obtained as brownish powders.

**Compound 1**: Yield: 21 % from **L1**. HPLC(Gradient 1): *R*_t_=3.13 min; ^1^H NMR (400 MHz, CDCl_3_): *δ*=2.70 (m, 1 H), 4.59 (d, *J*=1.6 Hz, 2 H), 5.21 (d, *J*=17.7 Hz, 2 H), 5.50 (d, *J*=17.7 Hz, 2 H), 7.66 (t, *J*=7.5 Hz, 2 H), 7.74 (d, *J*=8.3 Hz, 2 H), 7.82–7.91 (m, 2 H), 7.90 (d, *J*=8.0 Hz, 2 H), 8.35 (d, *J*=8.2 Hz, 2 H), 8.49 ppm (d, *J*=8.8 Hz, 2 H); ^13^C NMR (125 MHz, CDCl_3_): *δ*=56.86, 69.07, 76.49, 79.62, 120.40, 128.32, 128.68, 129.70, 133.11, 141.55, 147.13, 164.93, 193.91, 195 ppm; HRMS‐ESI (CH_3_CN/H_2_O): *m*/*z* calcd for [C_26_H_20_N_3_O_3_Re]: 609.1057; found: 609.1077.

**Compound 2**: Yield: 11 % from *N*‐Boc‐1,2‐diaminoethane. HPLC(Gradient 1): *R*_t_=3.03 min; ^1^H NMR (400 MHz, CDCl_3_): *δ*=3.04–3.07 (m, 1 H), 3.17–3.24 (m, 1 H), 3.46–3.48 (m, 1 H), 3.81–3.83 (m, 1 H), 4.56–4.58 (m, 1 H), 4.96–5.08 (m, 3 H), 5.42–5.45 (m, 1 H), 6.64–6.70 (m, 1 H), 7.45 (d, *J*=8.3 Hz, 1 H), 7.58 (d, *J*=8.3 Hz, 1 H), 7.65–7.68 (m, 2 H), 7.81–7.88 (m, 2 H), 7.91–7.92 (m, 1 H), 7.95–7.97 (m, 1 H), 8.14 (d, *J*=8.4 Hz, 1 H), 8.33 (d, *J*=8.34 Hz, 1 H), 8.38 (d, *J*=8.34 Hz, 1 H), 8.60 ppm (d, *J*=8.8 Hz, 1 H); ^13^C NMR (125 MHz, CDCl_3_): *δ*=45.96, 56.42, 68.09, 68.76, 77.15, 110.15, 119.41, 123.15, 127.66, 128.50, 128.96, 129.69, 130.76, 133.10, 137.77, 141.49, 147.92, 153.47, 161.99, 193.81, 194.66, 195.60 ppm; HRMS‐ESI (CH_3_CN/H_2_O): *m*/*z* calcd for [C_25_H_22_N_4_O_3_Re]: 613.1245; found: 613.1294.

**Compound 3**: Yield: 28 % from **L3**. HPLC(Gradient 1): *R*_t_=3.20 min; ^1^H NMR (400 MHz, CDCl_3_): *δ*=1.10 (t, *J*=7.3 Hz, 3 H), 2.11–2.15 (m, 2 H), 3.78–3.81 (m, 2 H), 5.00–5.03 (m, 2 H), 6.15–6.18 (m, 2 H), 7.60–7.63 (m, 2 H), 7.77–7.85 (m, 2 H), 7.83–7.85 (m, 2 H), 7.94 (d, *J*=8.43, 2 H), 8.29 (d, *J*=8.42, 2 H), 8.45 ppm (d, *J*=8.8 Hz, 2 H); ^13^C NMR (125 MHz, CDCl_3_): *δ*=11.39, 20.48, 69.06, 70.82, 121.04, 128.20, 128.43, 128.59, 129.65, 132.87, 141.38, 165.25, 194.52, 195.92 ppm; HRMS‐ESI (CH_3_CN/H_2_O): *m*/*z* calcd for [C_26_H_23_N_3_O_3_Re]: 611.1291; found: 612.1292.

**Bacteria strains**: Control strains of *Escherichia coli* (ATCC 25922) and *Staphylococcus aureus* (ATCC 25923) were used to determine the activity of the rhenium complexes. Methicillin resistant *S. aureus* (MRSA, ATCC 43300) and colistin resistant *E. coli* (mcr‐1, faecal clinical isolate^[23]^) were used to assess if **1** holds its activity against these strains. To characterise the cellular uptake of **1**, three *E. coli* mutants (MB4902, *lpxC* outer‐membrane permeable strain; MB5747, *tolC* efflux‐negative strain; and MB5746 *lpxC* and *tolC* permeable efflux‐negative) and their wild‐type parent strain (MB4827) were used.^[15]^ The mutant *tolC*, *lpxC* and *tolC* + l*pxC E. coli* strains MB4902, MB5747, MC5746 and control strain MC4827 were generously supplied by Merck Sharp & Dohme (Kenilworth, NJ). The mcr‐1 *E. coli* strain was generously provided by Dr. Bone Siu‐Fai Tang from the Hong Kong Sanatorium & Hospital. *Pseudomonas aeruginosa* mutants (PAO1, PAO750, PAO397) were kindly provided by Herbert Schweizer at Colorado State University.^[19]^

**Minimum inhibitory concentration (MIC)**: Bacteria were cultured in Cation adjusted Mueller Hinton broth (CAMHB; BD, Cat. # 212322) at 37 °C overnight with shaking (ca. 200 rpm). A sample of each culture was then diluted 40‐fold in fresh CAMHB and incubated at 37 °C for 1.5–3 hours with shaking (ca. 200 rpm). Compound stock solutions were prepared as 1.28 mg mL^−1^ in water or 20 % DMSO. The compounds were serially diluted two‐fold across the wells of 96‐well plates (Polystyrene, Corning, catalogue No. 3370) in quadruplets. Mid‐log phase bacterial cultures were added to a final cell density of 5×10^5^ colony forming units (CFU) mL^−1^ to the compound containing plates and immediately irradiated (1 hour at 365 nm) or kept in the dark. We found that the results of the assay were influenced by a number of factors. Using 384 well plates resulted in higher (less potent) MICs. Removing the lid covering the plates gave better results, probably due to light absorption, that is, arranged parallel approximately 6 cm under the light tube. Finally, the orientation of the well and distance from the lamp was optimised to obtain the best results. After treatment they were incubated at 37 °C for 18–24 h. MICs were determined visually as the lowest compound concentration at which no bacterial growth was visible.

To study the effect of different antibiotics in combination with the rhenium metal complex, the same procedure was followed with the addition of a fix sub‐MIC concentration (1/4 MIC) of polymyxin B (Sigma, P0972), octapepetin C4 (WuXi), meropenem (Sigma, Y0001252) and gentamicin (Sigma, G1914).

**Cell viability assay for 1**: Cytotoxicity to HEK‐293 (ATCC® CRL‐1573) human embryonic kidney epithelial cells was determined using resazurin assay. Briefly, cells suspended in DMEM medium (Gibco; 11330032) (supplemented with 100 U mL^−1^ each Penicillin/Streptomycin (Invitrogen; 15070063) and 10 % FBS (GE; SH30084.03)), were seeded into a 96‐well black‐walled clear‐bottom tissue culture plates (Corning, 3603) at 15 000 cells per well and incubated for ca. 20 hours at 37 °C, 5 % CO_2_.

After the incubation, compound **1** was added to cells and the plate was irradiated at 365 nm for 1 hour without a lid or incubated in the dark at RT. Plates were then incubated for another ca. 20 hours at 37 °C, 5 % CO_2_. Resazurin (Sigma; R7017) was added to cells (final concentration 10 μm) and plates were incubated for 3–4 hours at 37 °C, 5 % CO_2_ before measuring fluorescence intensity using the TECAN Infinite M1000 PRO with excitation/emission 560/590 nm. The data were analysed using GraphPad Prism and cell viability was calculated using Equation 1:

(1) $$\mathrm{Cell}\;\mathrm{viability}\;\left(\%\right)=\left(\mathrm{FI}{}_{\mathrm{Sample}}-\mathrm{FI}{}_{\mathrm{Negative}}/\mathrm{FI}{}_{\mathrm{Untreated}}-\mathrm{FI}{}_{\mathrm{Negative}}\right)\times 100$$

**Rhenium complex accumulation in bacteria**: Bacteria were cultured in Luria–Bertani broth (LB; Difco, Cat. #244620) at 37 °C overnight with shaking (ca. 200 rpm). A sample of each culture was the diluted 40‐fold in fresh LB and incubated at 37 °C shaking (ca. 200 rpm) until mid‐exponential (OD_600_=0.6, which corresponds to ca. 0.6×10^9^ CFU mL^−1^) phase was reached. The cells were then harvested at 3000 g for 15 min at 20 °C and resuspended in phosphate buffered saline (PBS) to OD_600_=6 (ca. 6×10^9^ CFU mL^−1^). Washed cells were transferred to a glass vial containing compound (final concentration of 50 μm) or buffer, and incubated for 60 min in water bath at 37 °C. After incubation, the cells were spun down (18 000 g, 5 min) and the supernatant was collected. Lysing buffer (0.1 m glycine‐HCl buffer, pH 3) was added to the pellet, mixed well, and incubated overnight at RT. After lysis, the tubes were centrifuged (18 000 rpm, 5 min) to separate cell debris (pellet) from cytoplasmic and periplasmic content. The three separate samples: supernatant after incubation (supernatant), supernatant after lysis (Lysate), and cell debris after lysis (pellet). All samples were freeze dried to remove all remaining solvents. Ultrapure nitric acid (200 μL for pellet and lysate and 500 μL for supernatant) was added and the Eppendorf vials were shaken at 40 °C for 24 h. The samples were then diluted (dilution was adjusted so that final rhenium content would not be higher than 30 ppb due to detection limits of the instrument) to a final volume of 10 mL and a final HNO content of 2 % before ICP‐MS measurement of rhenium content. The rhenium distribution is given as the average of two samples for each fraction.

**ICP‐MS measurements**: ICP‐MS experimental work was performed at the Environmental Geochemistry Laboratory of the School of Earth and Environmental Sciences, The University of Queensland.

## Conflict of interest

The authors declare no conflict of interest.

## Supporting information

As a service to our authors and readers, this journal provides supporting information supplied by the authors. Such materials are peer reviewed and may be re‐organized for online delivery, but are not copy‐edited or typeset. Technical support issues arising from supporting information (other than missing files) should be addressed to the authors.

Supplementary
