# Supplementary material for: Light‐Activated Rhenium Complexes with Dual Mode of Action against Bacteria
Source: Chemistry. 2020 Jan 30;26(13):2852–8. doi: 10.1002/chem.201904689 (PMC7687258; doi:10.1002/chem.201904689)
Supplement: Supplementary file 1 — Supplementary [file CHEM-26-2852-s001.pdf]

CHEMISTRY
A European Journal

Supporting Information

Light-Activated Rhenium Complexes with Dual Mode of Action against Bacteria

Angelo Frei,* Maite Amado, Matthew A. Cooper, and Mark A. T. Blaskovich*[a]

chem_201904689_sm_miscellaneous_information.pdf

Experimental

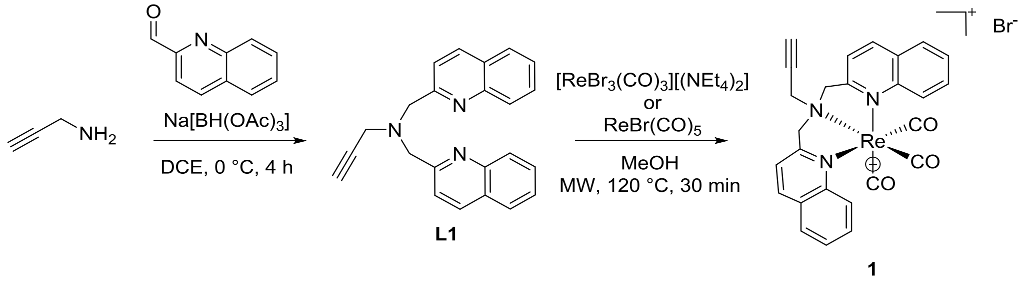

Scheme S1. Synthetic route to **L1** complex **1**.

Ligand **L1** was prepared according to reported procedure and purified by chromatography on silica (3:1 Hexane/EtOAc) as reported.[1]

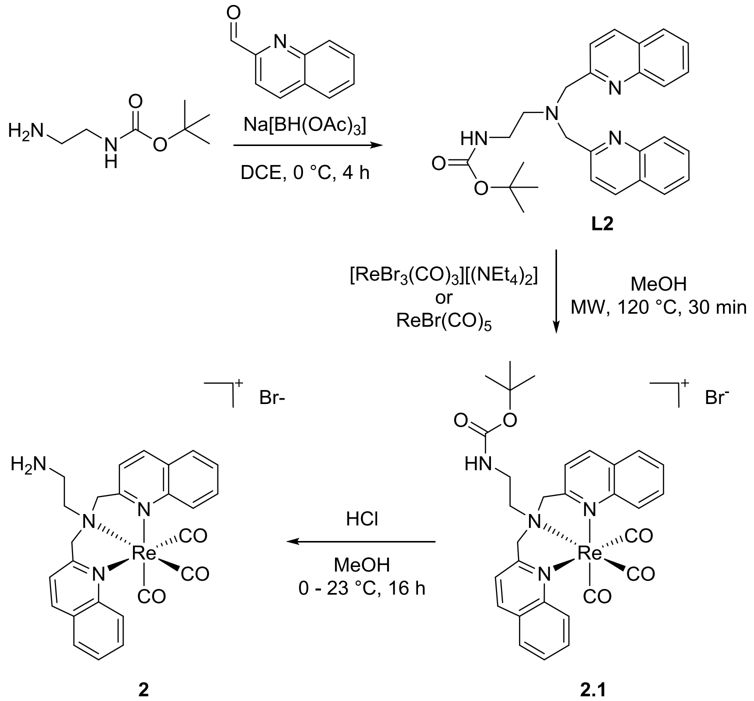

Scheme S2. Synthetic route to **L2** and complex **2**.

N-Boc-1,2-diaminoethane was prepared as reported previously.[2] **L2** was prepared using an analogous procedure as for **L1** and used without further purification to prepare **2**.**1** according to the general procedure for rhenium complexes.[1] The crude of **2.1** was dissolved in MeOH (2 ml) and cooled by means of an ice bath. Then 35% HCl (1 ml) was slowly added. The next day, the pH was adjusted to 12 with NaOH and the solvent was evaporated. The solids were taken up in

MeOH to remove excess NaCl and evaporated again after filtration. The crude of **2.1** was then purified by preparative HPLC (method A).

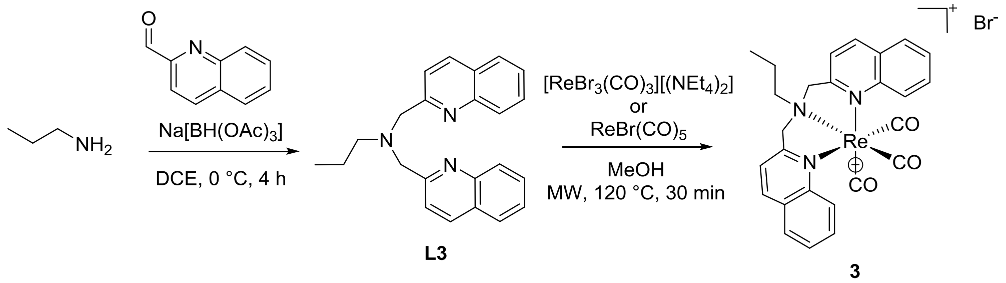

Scheme S3. Synthetic route to **L3** and complex **3**.

L3 was prepared using an analogous procedure as for **L1** and then used without further purification for the preparation of **3** according to the general procedure for rhenium complexes.[1]

ICP-MS measurements.

ICP-MS experimental work was performed at the Environmental Geochemistry Laboratory of the School of Earth and Environmental Sciences, The University of Queensland.

Analysis

Analysis by ICP-MS (Agilent 7900) is done in collision mode through the He collision cell. Instrument parameters are as follows: RF power: 1550 W, Carrier gas: 1.08 L/min, Nebuliser pump: 0.10 rps He Flow: 5 mL/min, ^{115}In was used as an internal standard to monitor instrumental drift during the experiment. The ICP-MS is a highly sensitive instrument and easily damaged. Its working range is around 10 ppb, and concentrations above 30 ppb should be avoided.

Experimental Procedure

Aqueous samples are arranged in auto-sampler tubes in a numbered rack in the auto-sampler. Calibration standards are prepared to enclose the range of expected concentrations and also loaded into the auto-sampler. A separate set of standards are made up from different primary standards to that used for preparing the calibration standards, to serve as controls for the calibration and the instrument performance. These are used to test the **accuracy** of the analytical process, designated as the **standards as unknowns**. Instrument blanks (that is, solvent blanks consisting of 2% ultrapure nitric acid) are analysed at regular intervals during the experiment to determine the detection limits for the experiment. At least 7 blanks are required for a good detection limit determination. The internal standard is automatically added to each sample during analysis to correct for internal drift. A monitoring sample, similar to the samples being analysed, is also analysed at regular intervals during the experiment, to monitor any unexpected

variations in instrument performance (external drift) and used to correct for this. Duplicate analysis are taken of every 10th sample at the end of the experimental run, to determine precision of analysis.

Data Reduction

Data reduction is completed after exporting the raw data to Excel and completing drift corrections, calibration and subsequent calculations in Excel. The data is processed as follows:

1. Export the raw data from the instrument to a csv or Excel file.

2. Pre-treat the data. Pre-treatment may include transformation, blank subtraction and internal and external drift correction. The degree of pre-treatment is dependent on the software associated with the analytical instrument.

3. Construct calibration curves for the analysed data, using the known concentrations of the standards and the instrument response to each analyte.

4. Determine whether the calibrations are linear or non-linear. Accurate calculations can only be done on linear calibrations. If necessary, remove the most concentrated standards from the calibration line until a linear result is achieved.

5. Determine whether the analyte content for all samples fall within calibration range. If there are analytes beyond calibration range, the analysis will have to be repeated, using either a more concentrated standard or a more diluted sample. For ICP-MS, a more diluted sample is generally required.

6. Once suitable calibration figures have been established, test the legitimacy of the calibration by calculating the concentrations of the calibration standards from the intensity data and the newly-constructed calibration curves. The concentration of the standards should be within 90-110% of the actual, known concentrations. For values in the ppb range, concentrations within 80%-120% of the known value, are acceptable. If not, the calibration curves will need to be refined until acceptable values are obtained. This step is known as recovery.

7. Calculate the standards as unknowns. Their values should be within 5-10% of their expected value.

8. Calculate the detection limits by finding the standard deviations of the intensity values for the instrument blanks and multiplying the value with 3. Then calculate the associated concentration (the detection limit) by using this value and the calibration equation for each analyte.

9. Calculate the precision or reproducibility of the analysis by calculating the average and standard deviation on each set of duplicates. Multiply the standard deviation with 100 and divide

by the average. This figure is the percentage relative standard deviation. For trace elements, they should not exceed 5%.

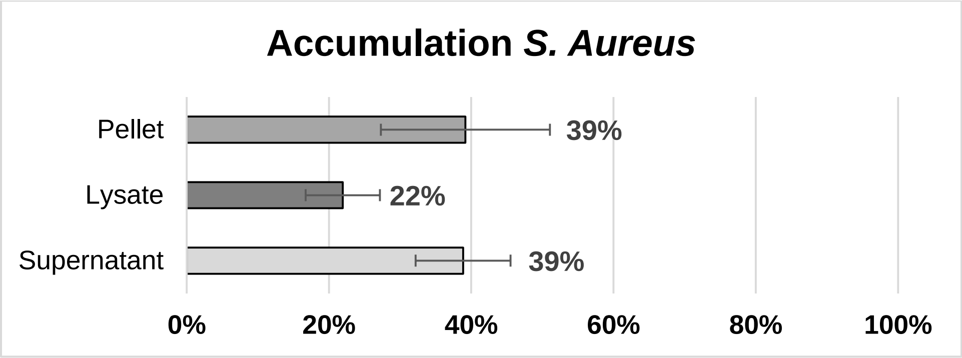

Figure S1. Cellular uptake and distribution of rhenium in *S. aureus* after 60 min (incubated with 50 µM of **1**). Percentage of rhenium in different fractions given as percentage of total detected rhenium.

Spectral Data for Complexes 1-3.

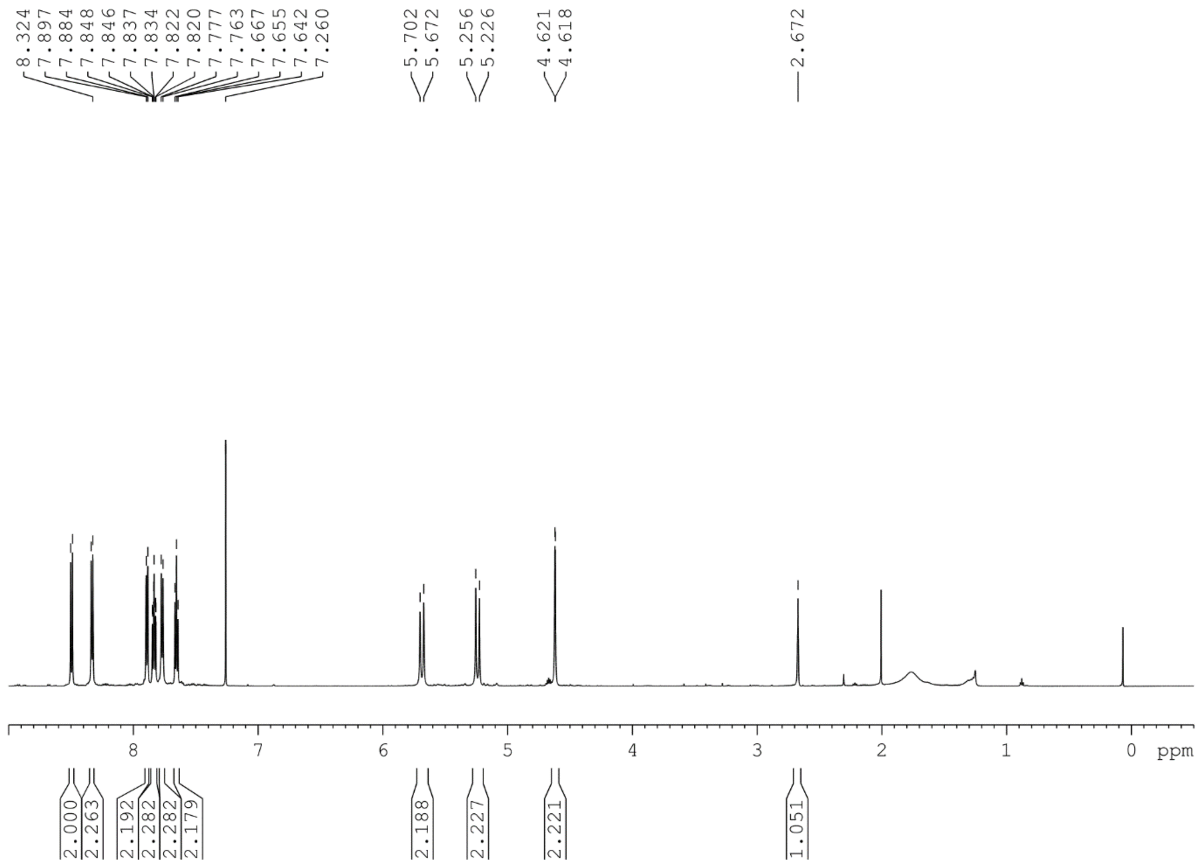

Figure S2. ^{1}H NMR of Compound **1** in $CDCl_3$.

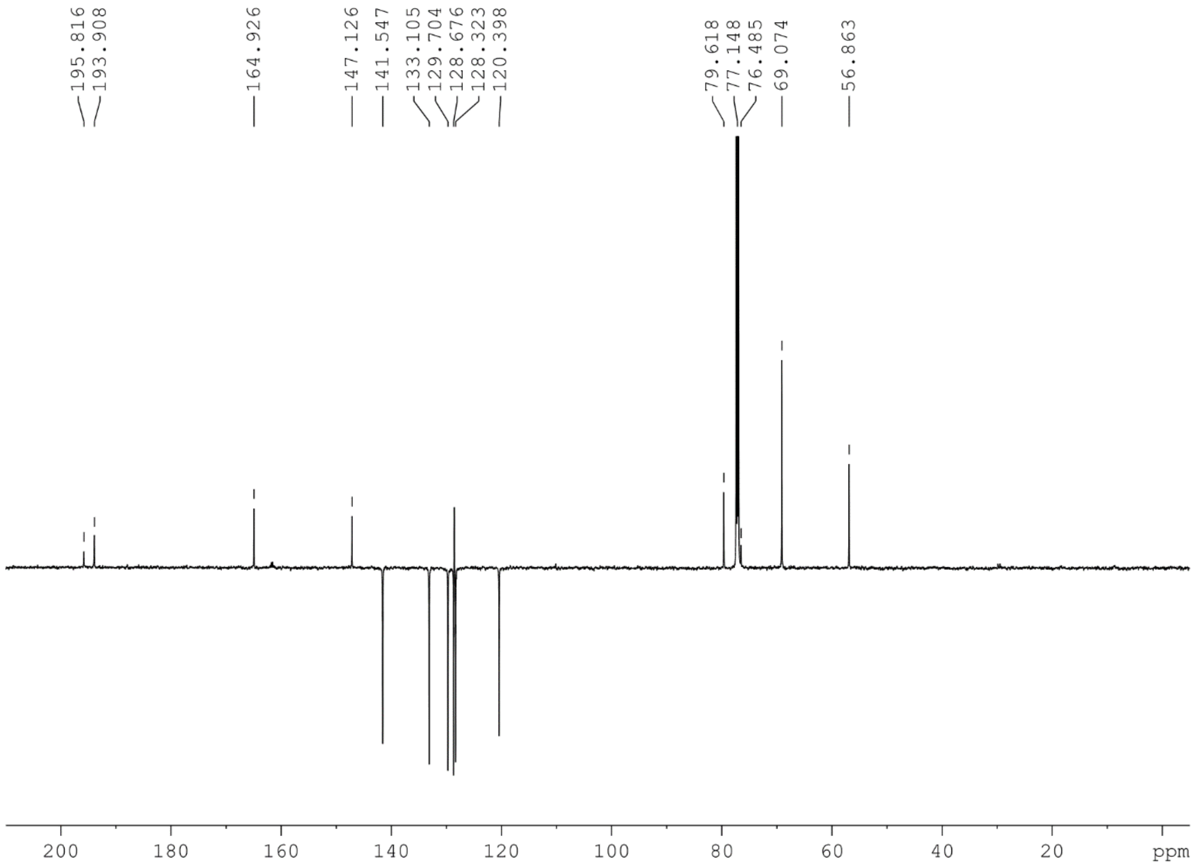

Figure S3. ^{13}C NMR of Compound **1** in $CDCl_3$.

Mass Spectrum SmartFormula Report

Analysis Info

		Acquisition Date	8/2/2018 3:02:01 PM
Analysis Name	D:\Data\cooper\MCC009327_DJE_02_08_2018_RB2_01_13197.d		
Method	tune-wide_50ul_hystar_withcal_direct_medmassde_2.m	Operator	a.piggott
Sample Name	MCC009327_DJE_02_08_2018	Instrument	micrOTOF 213750.00232
Comment	Comments		

Acquisition Parameter

Source Type	ESI	Ion Polarity	Positive	Set Nebulizer	0.8 Bar
Focus	Not active			Set Dry Heater	180 °C
Scan Begin	100 m/z	Set Capillary	4500 V	Set Dry Gas	5.0 l/min
Scan End	1000 m/z	Set End Plate Offset	-500 V	Set Divert Valve	Source

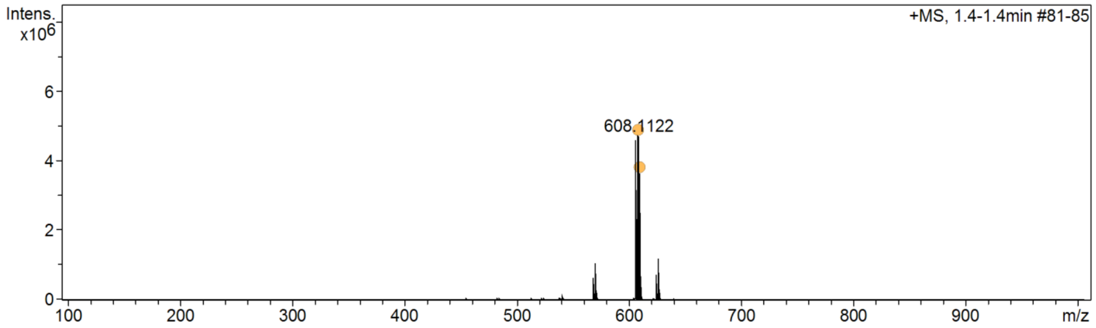

Figure S4. HR-ESI MS of compound **1** (H_2O/CH_3CN).

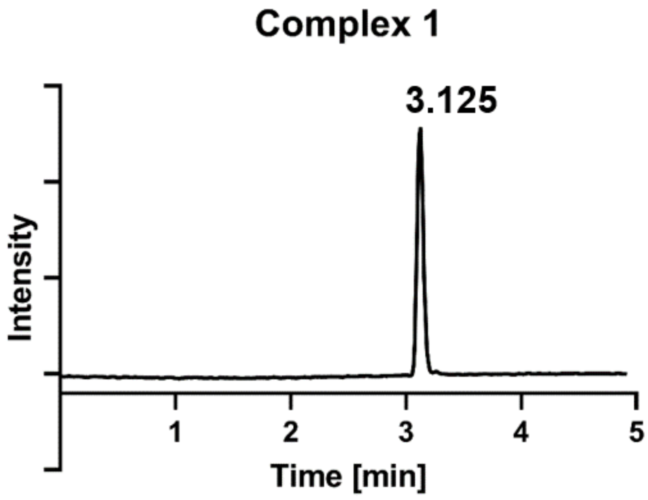

Figure S5. HPLC trace for complex **1** at 254 nm.

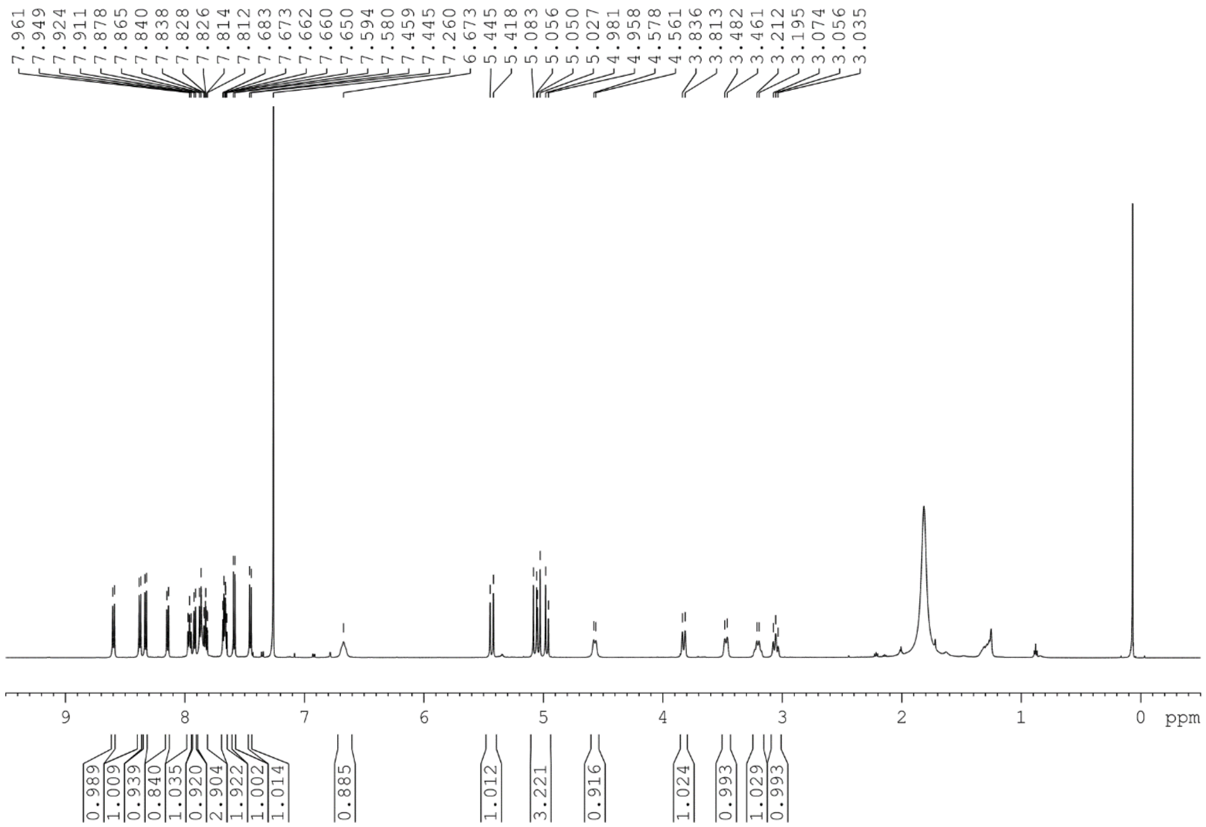

Figure S6. ^{1}H NMR of Compound **2** in $CDCl_3$.

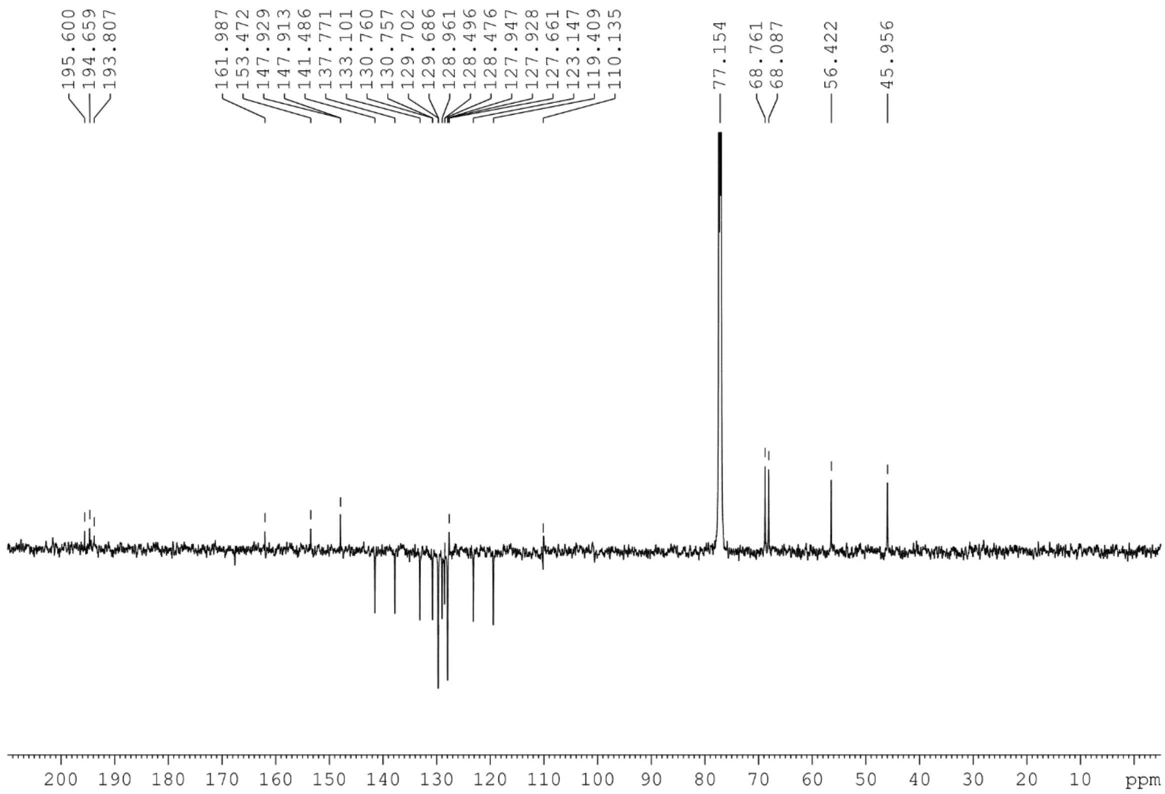

Figure S7. ^{13}C NMR of Compound **2** in $CDCl_3$.

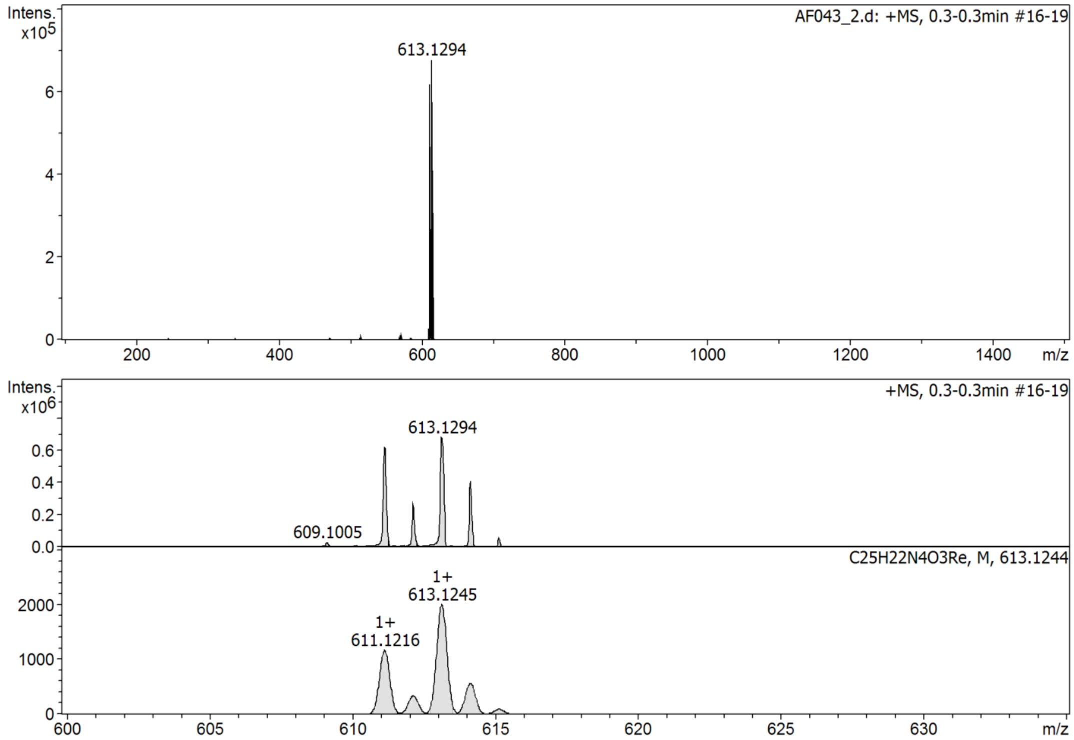

Figure S8. HR-ESI MS of compound **2** (H_2O/CH_3CN).

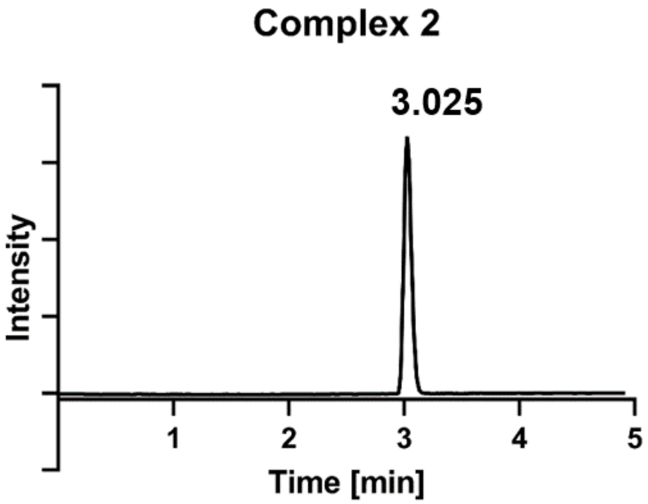

Figure S9. HPLC trace for complex 2 at 254 nm.

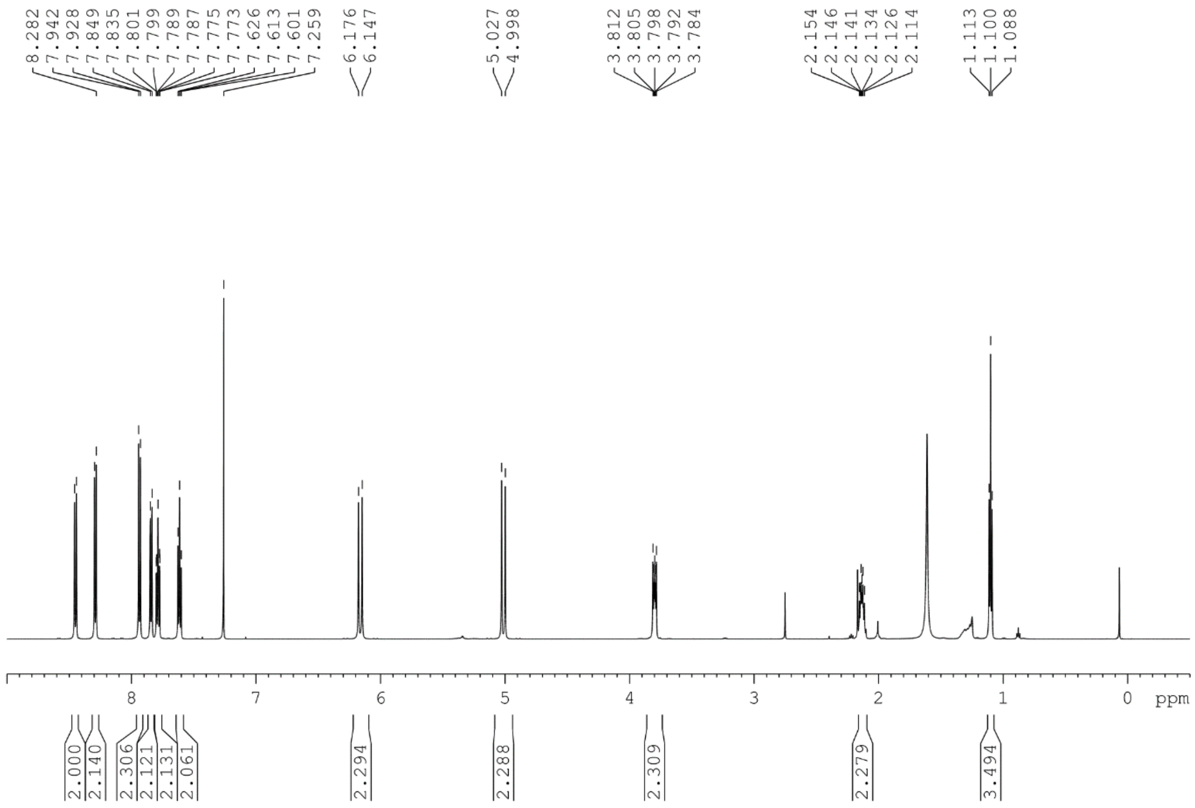

Figure S10. ^{1}H NMR of Compound **3** in $CDCl_3$.

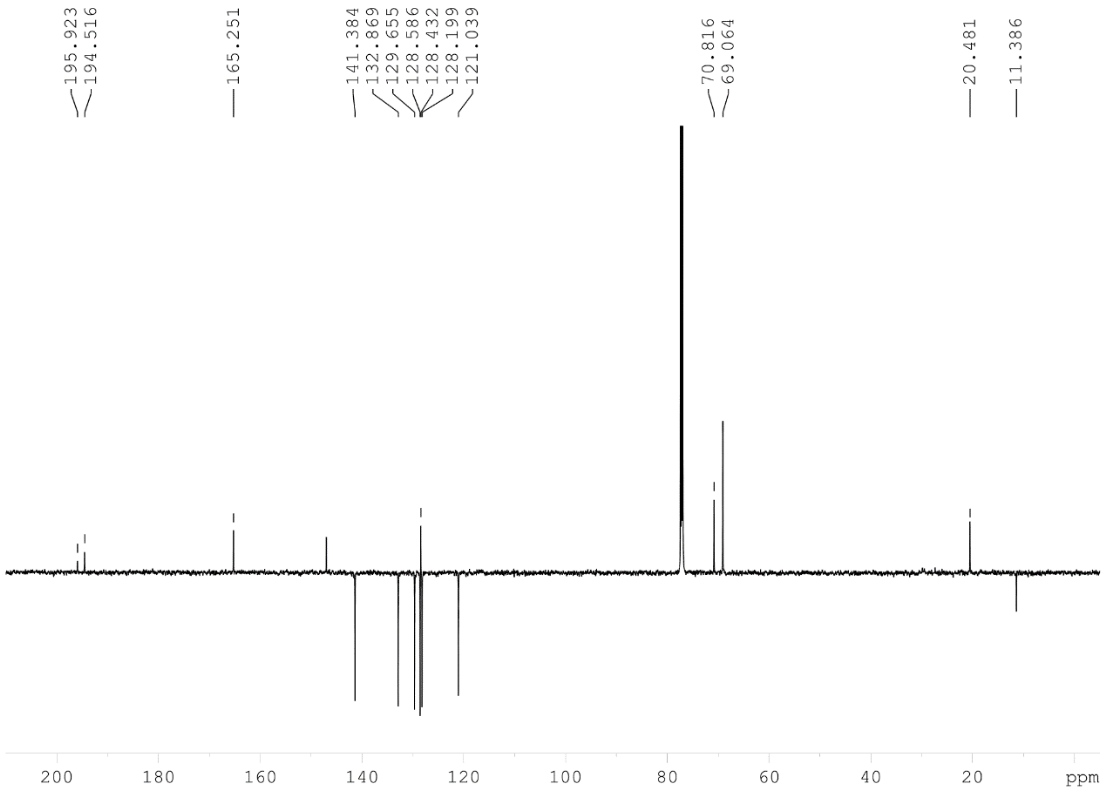

Figure S11. ^{13}C NMR of Compound **3** in $CDCl_3$.

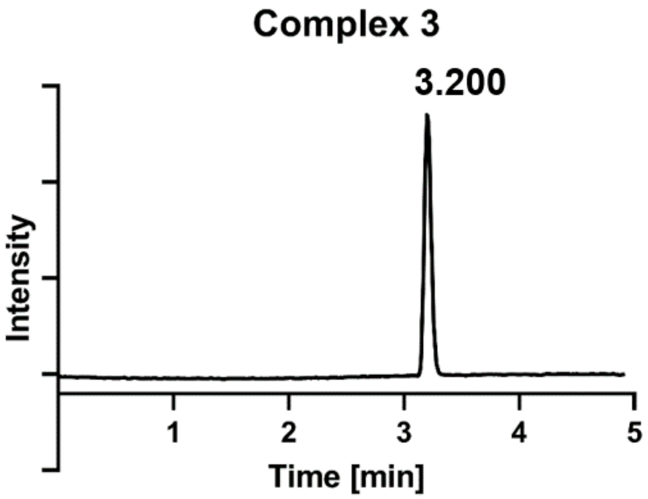

Figure S12. HPLC trace for complex **3** at 254 nm.

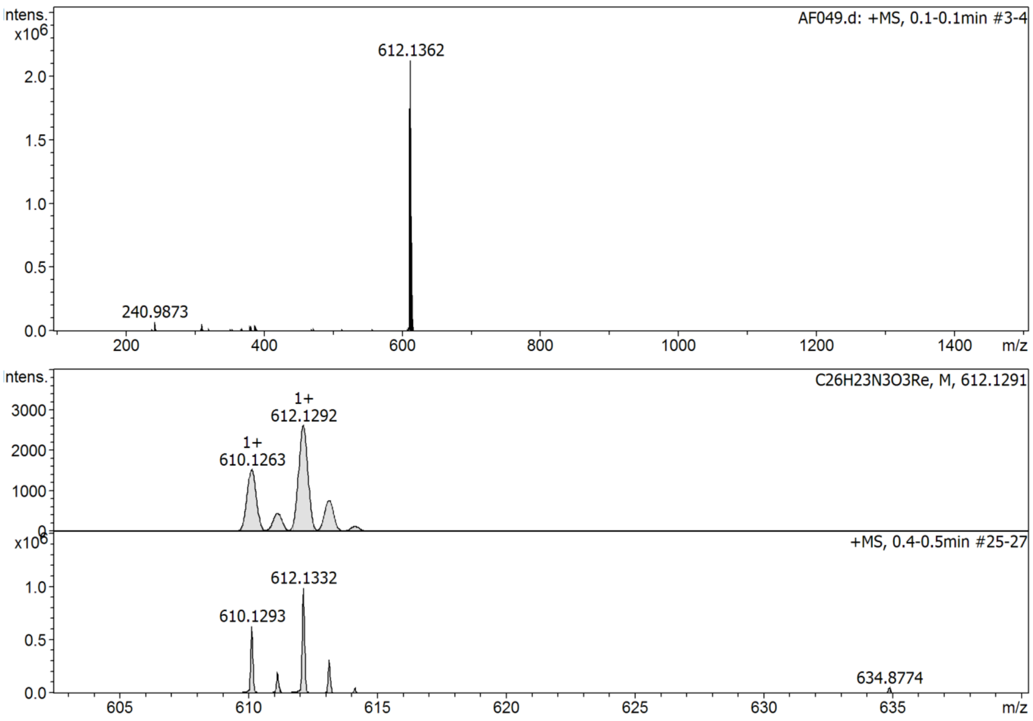

Figure S13. HR-ESI MS of compound **3** (H_2O/CH_3CN).

Table S1

MICs against Gram(-)

	dark		365 nm	
	K. pnemoniae (ATCC700603) MDR			
	[µg/ml]	[µM]	[µg/ml]	[µM]
**Pmx**[a]	0.25	0.2	0.25	0.2
**1**	>128	>186.2	128	186.2
	A. baumannii (ATCC19606)			
	dark		365 nm	
	[µg/ml]	[µM]	[µg/ml]	[µM]
**Pmx**[a]	0.25-5	0.2-0.4	0.125-0.25	0.1-0.2
**1**	128	186.2	16-32	23.1-46.2

Cell Viability Assay Compounds 2 and 3

Cytotoxicity to HEK-293 (ATCC® CRL-1573) human embryonic kidney epithelial cells was determined using resazurin assay. Briefly, cells suspended in DMEM medium (Gibco; 11330032) (supplemented with 100U/mL each Penicillin/Streptomycin (Invitrogen; 15070063) and 10% FBS (GE; SH30084.03)), were seeded into a 384-well black-walled clear-bottom tissue culture plates (Corning,) at 5000 cells per well.

Serially diluted compounds were added to plate containing cells and incubated for ~20 hours at 37 °C, 5% CO_2.

After the incubation, resazurin (Sigma; R7017) was added to cells (final concentration ~10 µM) and plates were incubated for 3-4 hours at 37 °C, 5% CO_2. The fluorescence intensity was read using the TECAN Infinite M1000 PRO with excitation/emission 560/590 nm. The data was analyzed using GraphPad Prism and cell viability was calculated using the following equation: Cell viability (%) = (FI_{SAMPLE} – $FI_{Negative}$/$FI_{UNTREATED}$ –$FI_{Negative}$)*100.

Haemolysis Assays

Human whole blood (Australian Red Cross) was washed three times with 3 volumes of 0.9% NaCl and resuspended in a concentration of 0.5 x 10^8 cells/mL, determined by manual cell count in a Neubauer haemocytometer. Washed cells were added to compound containing plates (384-well polypropylene plates (PP); Corning 3657) for a final volume of 50 µL, shaken and incubated for 1 h at 37 °C. After incubation, the plates were centrifuged at 1000 g for 10 min to pellet cells and debris, 25 µL of the supernatant was then transferred to reading plates (384-well, polystyrene plated (PS), Corning CLS3680), with haemolysis determined by measuring the supernatant absorbance at 405 mm (OD405), using cells without inhibitors as negative control and cells with 1% Triton X-100 (Sigma T8787) as positive control. HC_{10} and HC_{50} (concentration at 10% and 50% haemolysis, respectively) were calculated by curve fitting the inhibition values vs. log(concentration) using a sigmoidal dose-response function with variable fitting values for top, bottom and slope. Melittin (Sigma M2272) was used as internal control on each plate.

**Compound**	**CC_{50} [µM]**	**HC_{10} [µM]**
**1**	38.1 ± 0.5	>300
**2**	97.7 ± 8.8	>300
**3**	14.9 ± 0.1	139.5 ± 0.1

Table S2. Cytotoxicity (CC_{50}) and Haemolysis (HC_{10}) values for compounds **1-3**, (determined in 384 well plates, whereas the dark/photo-toxicity for **1** mentioned in the main text was determined in 96 well plates).

[1] E. Benoist, Y. Coulais, M. Almant, J. Kovensky, V. Moreau, D. Lesur, M. Artigau, C. Picard, C. Galaup, S. G. Gouin, *Carbohydr. Res.* **2011**, *346*, 26-34.

[2] D. Muller, I. Zeltser, G. Bitan, C. Gilon, *J. Org. Chem.* **1997**, *62*, 411-416.
